# Extraordinary Will Case, Involving the Question of Mental Capacity

**Published:** 1851-04-01

**Authors:** 


### EXTRAORDINARY WILL CASE, INVOLVING THE QUESTION OF MENTAL CAPACITY.

DRYDEN *v.* FRYER.—THE WILL OF THE LATE SIR GREGORY PAGE TURNER.

COURT OF QUEEN'S BENCH.—Sittings at Nisi Prius at Westminster.
Before Lord Campbell and a Special Jury.

Mr. M. Chambers, Q.C., Mr. Peacock, Q.C., and Mr. Rickards, were counsel for the plaintiff; and the Solicitor-General, Sir F. Thesiger, Q.C., Sir F. Kelly, Q.C., and Mr. Barstow, represented the defendant.

This was an issue directed by the Court of Chancery to try whether the late Sir Gregory Page Osborne Turner, a baronet, was in a sound state of mind on the 15th of June, 1841, upon which day he made his will. The plaintiff was the first cousin of the late Sir G. P. Turner, and the defendant was the brother of the Rev. Charles Gulliver Fryer, who had married the only daughter of the testator. The plaintiff was one of the executors of the will, and had a legacy left him for his trouble, whilst the defendant, as we understood, was not affected by the will in any way. The reason why these parties were selected to be plaintiff and defendant was, because there was a clause in the will to the effect that if any of the parties beneficially interested in it should dispute it, their interests would thereby become affected. Sir G. P. Turner had twice been declared a lunatic—once in 1814, when a commission of lunacy had issued against him, and again in 1823, when a fresh commission was issued. The first commission was set aside in 1815, but the second never was, although about the year 1840 all restraint was removed from the testator, on the ground, as the plaintiff alleged, that he had recovered his senses. The details of the case will be best understood from the evidence, which we proceed to give fully. The first witness called was—

Mrs. Helen Eliza Chumley, examined by Mr. Peacock.—Witness was the widow of the late Sir Gregory Page Turner, and had married again. Was married to the testator in 1818. Had known the late Sir Gregory Page Turner ever since he was seventeen years of age. His father died about the year 1806. The testator was in a good state of mind when witness married, but he was always an eccentric man. Witness had one surviving daughter by the testator, and a son who had died. The daughter was born in 1820. Witness was appointed one of the committee of the testator's person, when he was declared a lunatic. Witness recollected the marriage of her daughter, in 1838, with the Rev. Mr. Fryer. Before the marriage, Mr. Fryer had engaged to settle 20,000*l.* upon Miss Turner—a Mr. Maberley was, at first, employed as the solicitor in the matter. Mr. Fryer wanted to substitute some other property instead of the 20,000*l.* in money, to which witness objected; but Mr. Fryer still persisted in his plan; and as witness's daughter would be married to him, witness went to another solicitor, a Mr. Barney, who lived at Southampton. Mr. Fryer and her daughter went with her, and Mr. Barney drew up a marriage settlement. Witness's daughter was married on the 22nd of August, 1838. The family seat of Sir G. P. Turner was called Battlesden House, and was situated in Bedfordshire. In the year 1838 it was very scantily furnished. Mr. Fryer furnished some rooms in the house, and went to reside there with his wife. The testator was angry at that being done. Mr. Maberley wrote several letters to Mr. Fryer, expressing Sir G. P. Turner's displeasure, and Mr. Fryer and his wife then left. In the year 1823 two keepers were appointed to live with the testator, and witness at that time left him. A commission of lunacy had been issued against him in November, 1823. After some time the testator went to reside in the Alpha-road, Regent's-park. Sir G. P. Turner's state of mind improved from about the year 1840. On the 7th of May, 1840, witness returned to live with him, and lived with him until he died, which was in 1843. His mind continued to improve, and he talked very rationally. In consequence of the improvement of Sir G. P. Turner's mind both his keepers were removed from him. On the 6th of February, 1841, all restraint was removed from him. In November, 1840, Sir G. P. Turner dictated a paper to witness. The paper now produced was the one in question, and was written by witness. A paper was then put in and read, in which the late Sir Gregory Page Turner stated that it was his intention to leave all his property which he could dispose of to his daughter and her children, if she should have any, and his wife's jointure of 2000*l.* a year was to be chargeable on the property. The amount of witness's jointure was 2000*l.* a year. In 1841, when witness's daughter came of age, she came to visit her father and witness. A conversation took place between them and Mrs. Fryer, in which both Sir G. P. Turner and witness urged Mrs. Fryer to say, when she went before the Master of the Rolls, that she wished to have a settlement made upon her. She replied that she would say that she wished for a settlement, but would not go against the wishes of her husband. Witness did not see Mrs. Fryer but once after the conversation she had alluded to for a long time. In 1842 Sir G. P. Turner had a very bad epileptic fit. He had had epileptic fits before that time. After May, 1842, the fits became more violent, and a Mr. Rook, one of Sir G. P. Turner's keepers, came back to sleep in the room with him, in order to render him assistance in case he had a fit. After a time, Rook stayed with the testator in the day-time, as well as at night. In June, 1841, Sir G. P. Turner desired witness to write a letter to Mrs. Fryer, and to say that he and witness should be at all times happy to see her, but declined visiting Mr. Fryer until his marriage settlements were satisfactorily settled. From the Alpha-road, Sir G. P. Turner and witness went to live at No. 1, Montagu-square, in July, 1841. After that Sir G. P. Turner and witness went, in January, 1842, to live at 104, Gloucester-place, Portman-square. Witness's husband told her he had made his will, and effectually taken care of his daughter. Sir G. P. Turner kept his bedroom from October, 1842, until the time when he died. In witness's judgment, Sir G. P. Turner was capable, on the 15th of June, 1841, of performing a rational act. His will bore date on that day. He was then in the habit of talking rationally on all subjects.

Cross-examined by the Solicitor-General.—Witness was married again in August, 1844. Witness's present husband was the son of a surgeon living in Nottingham-place, New-road. In 1823 Sir G. P. Turner was in a state of pecuniary difficulty. After the commission of lunacy in 1823, when Sir G. P. Turner was found a lunatic, he went to the Queen's Bench Prison, and then to the Alpha-road. During that time witness saw him often. Witness lived with Mr. and Mrs. Fryer after their marriage. Witness lived with them at Battlesden, where they stayed about nine weeks. Sir G. P. Turner had considerable estates in Bedfordshire, Oxfordshire, and Kent. There was also property at Blackheath, Greenwich, and in London, Witness, her daughter, and Mr. Fryer, went to Battlesden for nine weeks in the year 1839. When witness went back, in 1840, to live with her husband, she had a serious misunderstanding with Mr. Fryer about the marriage settlement. On that occasion Mr. Fryer threatened that witness should not see her daughter any more if she (witness) did not act as he liked with respect to the marriage settlement. The house in the Alpha-rord belonged to a man named Holmes, but witness did not know that he was a professed lunatic keeper. Rook afterwards took the house when Holmes left it. A Mr. William Paxton was sent for by Sir Gregory in 1841. He had been steward to Sir Gregory's estates in Bedfordshire. Sir G. P. Turner was much attached to him. When witness returned to live with her late husband, he had lost all his strange fancies. He had never been guilty of such indecencies that witness could not live with him. Sir G.

P. Turner had one peculiarity, which was, that he would not shake hands with people he did not like. Witness had no doubt that he shook hands with many people in 1840. Witness had no doubt that he shook hands with the plaintiff, and also with his sister. Witness and a Mrs. Neale who had been her daughter's musical governess, was afterwards witness's amanuensis. Never told Sir G. B. Turner that, if he did not leave off his eccentricities, the commission of lunacy could not be superseded. In 1840 he did not sleep in bed in his clothes, as witness believed, or cut holes in his linen. Witness never saw him collect bits of waste paper and direct them to be preserved with care. Sir G. P. Turner used to call the commission of lunacy "a humbug." Witness never heard him say that he would bring an action against the Lord Chancellor on account of it. In 1840 witness contemplated superseding the commission, but never told Sir Gregory that if he did certain things it could not be superseded. Witness never told him that he must leave off doing certain things before strangers. He was very fond of his daughter. Witness stayed with Sir G. P. Turner in 1840, at his own request. Sir G. P. Turner's features became drawn one day whilst in witness's room, and it slightly affected his speech. The next day, however, he was as well as ever. Did not remember what salary she paid Rook. He left Sir G. P. Turner in February, 1841, and came back in October. During this interval Rook did not watch Sir G. P. Turner; but after May, 1841, he walked out with him on account of Sir Gregory having epileptic fits. The restraint was removed from Sir G. P. Turner, in order to try how he would behave, and he conducted himself so well, that witness thought the commission was in progress of being superseded. Witness supposed it was not done on account of the violence of Sir Gregory's epileptic fits. In 1841, application was made to the Court of Chancery to increase witness's allowance, which was increased to 2000*l.* a year. In February, 1841, Sir Gregory, witness, Mrs. Neale, W Paxton, King the coachman, Sutton the valet, and others, took an excursion into Bedfordshire. Sir Gregory used to wear very shabby clothes, but was clean in his linen. When this excursion took place, all the parties went to Luton, where Sir Gregory had a fit. After Sir Gregory left Luton he went into Cambridgeshire, and then returned to London. Sir Gregory then went to Brighton, but witness did not go with him, owing to indisposition. Sir Gregory then came back to London and went to Montagu-square. The house was taken for a year, but Sir Gregory only stayed there until January, 1842. The reason of his leaving was not because he believed the house was haunted by the ghost of the former occupier. The reason of leaving Montagu-square was, that witness thought the staircase was too near Sir G. P. Turner's bedroom, and she was afraid that in a fit he would fall down the stairs and kill himself. A servant said there had been a murder in the house. At this time Sir Gregory was in possession of his faculties, except when under the influence of the fits. Witness allowed Sir G. P. Turner to have a little pocket-money. He used to have 9*l.* or 10*l.* at a time, at his own request. Witness did give Sir Gregory's old clothes to an old servant, who lived at the St. Marylebone Almshouses. Witness had given a bond to indemnify the plaintiffs in the present action against the costs of the present proceeding. That was after Sir G. P. Turner's death. Mr. Fryer proposed to substitute 7000*l.* worth of tithe property as part of the 20,000*l.* to be settled on Miss Turner, instead of settling the whole sum in money upon her. Mr. Dryden was one of the committee on the person of Sir Gregory, and was his first cousin.

Re-examined by Mr. Peacock.—Witness's daughter had no family.

Mr. Joseph Maberley, examined by Mr. Rickards.—Witness was a solicitor of long standing. He and his father before him had been solicitors to Christ's Hospital. He and his father before him had also been solicitor to the late Sir Gregory Page Turner, and to his father. Witness had known the late Sir Gregory when he was a child. Witness had had opportunities of seeing Sir Gregory in various states of mind and fortune. Witness was present at the inquiry upon the first commission of lunacy against Sir Gregory, which was issued in 1814. Witness was privy to the proceedings which were taken to set aside that commission, which was done in 1815. Witness was solicitor to the second commission in 1823, and was present when Sir G. P. Turner was examined. The first commission was superseded by Lady Saye and Sele, the aunt of Sir Gregory. The second commission was issued by the late Sir E. P. Turner, brother of Sir Gregory. That commission was traversed, and witness was the solicitor who supported it. That commission was established. The medical gentlemen who attended Sir Gregory were Sir F. Milman, Dr. Warburton, and others. Witness always consulted with the medical men. Between the year 1815 and the year 1823

Sir Gregory was attacked with insanity in every year. It began with intoxication, which led on to direct lunacy. The attacks lasted a few weeks at a time. When they ceased his mind became right again. Whilst the attacks lasted, Sir Gregory was subject to personal restraint. Sir F. Milman always ordered when the restraint was to be used, and also directed when it was to be left off. Sir F. Milman was long since dead. Witness always interfered by Sir Gregory's request, who, on his recovery, always expressed his approbation of what witness had done. Sir Gregory's father died in 1805, and Sir Gregory came of age in 1806, and came into property worth then about 20,000*l.* a year. At that time Sir Gregory had very few associates of his own rank in life. He became acquainted with disreputable characters, who sought his society for their own gain. He was led by them into habits of intoxication. That was shortly before the first commission issued. When Sir Gregory was put under restraint, in 1814, his mind so soon improved that in the following year the commission was superseded, after Lord Eldon had seen Sir G. P. Turner. Soon after the first commission was superseded, Sir Gregory returned to his habits of intemperance, and continued them up to the year 1823. At all times Sir Gregory was an eccentric man. His father was also an eccentric man. Sir G. P. Turner was negligent in his person, and always regardless of personal appearance. He was shy and sullen towards strangers, and used to talk eagerly and in a very loud tone of voice. He was a man likely to produce an unfavourable impression upon strangers. Sir Gregory at one time of his life had a fear of infection, and if he saw a funeral he would turn away to avoid it. That peculiarity existed for a very long time. He never would shake hands with a person with his naked hand for fear of infection. He used also to stuff his trousers' pockets full of papers. Sir Gregory was a man of very great conversational talents, of considerable reading, and great information. He had been educated at Harrow and Oxford. He had a very good memory. His health began to give way about 1839 or 1840. At that period, however, his mind improved. Until 1839, Sir Gregory had always expressed a great dislike to his brother, Sir Edward Page Turner, and used to speak of him most contemptuously. He used to call him Captain Dallaway, which was the name of a butler in the family, whose son Sir Gregory said he was. On the 14th of August, 1839, Sir Gregory called upon witness, and told him that his brother Edward had been to see him, and said he was very much pleased at it, and desired witness to appoint his brother to meet him at witness's office. Witness did so. On the 17th of August, 1839, Sir Gregory and his brother met at witness's office. Sir Gregory addressed him very cordially, and appeared greatly pleased to see him, and they sat in friendly conversation together for about an hour. On the 18th February, 1841, Sir Gregory called upon witness alone, which was the first time witness saw him after his liberation from restraint. On that occasion he said to witness that he called to be congratulated on his liberation, and held out his naked hand and shook hands with witness. Witness had not known Sir Gregory for twenty years before to shake hands with people. On that occasion he opened the door with his naked hand, which it had never been his habit to do, for if he had not his glove on, he would wait for witness to open the door for him, and if witness did not do so, Sir Gregory would take the skirts of his coat in his hands, and so open the door. Sir Gregory had been averse to a medical man visiting him, but in the year 1839 that dislike entirely disappeared. Sir Gregory used continually to complain of the existence of the second commission, but would not allow the word "supersedeas" to be mentioned by witness, but insisted that it should be traversed a second time, it having been traversed once. He said the word "supersedeas" implied that the commission was right at the time it was issued, whereas a traverse denied that it was right at any time. In the year 1840, Sir Gregory wished to have a "supersedeas," but told witness that if the commission could not be superseded, he should be satisfied to be freed from personal restraint. Dr. Warburton and Dr. Southey directed the restraint to be removed. None of the delusions which witness had mentioned as having passed away from the mind of Sir G. P. Turner about the years 1839 or 1840 ever returned to his mind again. Witness received directions in July, 1838, from Lady Turner, to prepare a marriage settlement for Miss Turner. Miss Turner was then eighteen years of age, and Mr. Fryer applied to the Court of Chancery for permission to marry her. The matter was referred to the Master, who reported that the marriage was a proper one to take place. A Mr. Stevens acted as the solicitor for Mr. Fryer. The draft of the settlement was altered, and other properties were substituted for the 20,000*l.* Mr. Hodgson, the conveyancer whom witness had employed in the matter, said that other properties could not be substituted for the 20,000*l.* in money without the permission of the Court of Chancery. After the marriage, witness saw Mr. Fryer, and called upon him to act in accordance with the articles of agreement made at Southampton. He did not do so, and fifteen months after a bill in Chancery was filed. Mrs. Fryer was made a plaintiff to that bill by the direction of the Master of the Rolls. In February, 1841, Mrs. Fryer came of age, and disavowed the bill, and desired her name to be struck out as a plaintiff. The Master of the Rolls saw Mrs. Fryer, and on the 19th of April she went to court, and disavowed the bill herself. Witness saw the will now produced, signed by the late Sir G. P. Turner. It was dated 15th of June, 1841. Witness and Henry Elston, his clerk, were the attesting witnesses to it. Witness at that time thought Sir Gregory as competent to make a will as any man could be. The will was executed at witness's office. On the 22nd of April, 1841, Sir Gregory first mentioned to witness the subject of a will; that was at witness's office. On that day he gave witness generally to understand what his wishes were with respect to the disposition of his property. He said his great object was to protect his daughter. He appealed to witness, and said that he (witness) must see the necessity of doing so from what had taken place on the 19th of April, 1841. Whilst the Chancery proceedings were going on, Sir Gregory expressed great anxiety about them, and called often at witness's office. On the 29th April, Sir Gregory came again, by appointment, to witness's office. On that occasion a general conversation took place about the will. Sir Gregory came again on the 6th of May, and appointed to come again on the 13th. The draft of the will now produced was the draft which witness prepared. When Sir Gregory called on witness on the 13th, the draft was shown by witness to Sir Gregory, and was read over to him. Sir Gregory made his observations, and directed certain alterations and additions. Sir Gregory then appointed to come to witness's office on the 20th May, and desired witness to get the will of his father for the purpose of reference. Witness made a new draft, which was submitted to Sir Gregory on the 20th. Witness and Sir Gregory read the new draft over together, and when there was a passage which he did not understand, he would stop witness in order to have an explanation. On that occasion Sir Gregory directed further alterations. He had the name of Captain Bayfield, R.N., Lady Turner's brother, struck out as a trustee, as he said he was not a proper party, as he might be absent from England on service. Sir Gregory substituted the name of the Rev. Lempster Dryden, his cousin, the present plaintiff. Sir Gregory made another appointment with witness for the 27th of May. He came again on that day. The draft was again read over to him, and he suggested two alterations. One had reference to his executors. He wished that it should be expressed that the legacies which had been left them were left them "for their trouble herein," because a Mr. Stadpole, who was one of his father's executors had had his legacy disputed for not having acted, and Sir Gregory thought these words would prevent such a thing occurring again. On the meeting on the 13th of May, Sir Gregory directed a clause to be put in his will, that if any of his relations disputed the will, they should forfeit the interests they took under it. Sir Gregory then said to witness, that the situation in which he was gave opportunities for contesting his will. Sir Gregory, on the 27th of May, made another appointment for the 2nd of June. On that day he came and said the will was quite according to his wishes. He said, nevertheless, he would not sign then, but would call again for that purpose, so that anything which occurred to him to add, he might be able to add. On the 15th of June Sir Gregory came to execute the will, which he accordingly did. Witness was perfectly satisfied that he quite understood every provision in the will. Witness, several years before the will was made, had told Sir Gregory that he had made a will in which it was directed that all disputes were to be referred to the Attorney-General, or a barrister to be appointed by him, and Sir Gregory directed a similar clause to be inserted in his will. Sir Gregory directed his creditors to have interest upon their debts. He used to divide his creditors into two classes. He used to call his tradesmen his black sheep, and those who got about him to get money out of him by various means, his white sheep. Witness asked him if he meant his black sheep to have interest on their money as well as his white sheep? Sir Gregory seemed ashamed, and said to witness, "Don't talk so, sir—that's all gone by." Both classes of creditors were to have interest, by Sir Gregory's direction. There were several pictures which had belonged to the family which had been sold by the sheriff, and Sir Gregory having wished to have them back, and having made the pictures heir-looms in his family, witness asked him what he would have done about re-purchasing them, as he had once expressed a wish of that kind. Sir Gregory seemed rather offended with witness, and said he should like to have one or two that had belonged to his father, but nothing more. Witness knew nothing of the paper dictated to Lady Turner until after the death of Sir Gregory. Witness never went to Sir Gregory's house, and never saw him, but at his (witness's) office. The will was here put in and read. In it Sir Gregory left 2000*l.* a year to his daughter, 20,000*l.* to any one child she might have, 30,000*l.* if she had two, and 40,000*l.* if there were more, to be equally divided amongst them. If his daughter had no children, then the property which Sir Gregory could dispose of was left to his brother, Sir E. P. Turner, and his family. The will secured to the testator's widow her jointure of 2000*l.* a year. In July, 1839, Sir Gregory called on witness, and said he had understood that Mr. Fryer was going to live at Battlesden, and he objected to it, as it seemed as if he wished to take possession before his time. Witness was directed by Sir Gregory to write to Mr. Fryer about it. The next day Sir Gregory called on witness and showed him a newspaper which he had received from Mr. Fryer with the Woburn post-mark, and therefore he said he must be at Battlesden. Mr. Fryer appealed to the Master about living at Battlesden, who decided against it. The property left by Sir Gregory, after paying Lady Turner's jointure, and encumbrances, would be worth only 2000*l.* a year, although it had once been worth 20,000*l.* a year.

Cross-exmamined by the Solicitor-General.—After the death of Sir Gregory the entailed estates went to his brother, Sir Edward Page Turner. Witness raised money in 1816 for Sir Gregory. It was raised by granting annuities to the Eagle Assurance Company. The estates were encumbered to the extent of from 70,000*l.* to 90,000*l.* When Sir Gregory mortgaged his estates, witness was appointed the receiver, with five per cent. commission. Witness was solicitor to Sir Edward Turner, and was so now to his son, the present baronet. Witness made about 600*l.* a year by the receivership. The principal eccentricities which Sir Gregory was guilty of in 1823 were, that he cut holes in his bed sheets, and would not change his clothes. On one occasion the gardener had taken an old coat and cut it in shreds to nail up the fruit-trees, and Sir Gregory insisted on having the shreds back, and having them sewed together. He used to go to bed with his trousers under his pillow, and would not attend to the calls of nature. At that time witness had no doubt that Sir Gregory was mad. In 1839 Sir Gregory said to witness he should be satisfied if he could get the restraint taken off, and a little pocket-money allowed him. Mr. William Paxton held a farm on one of Sir Gregory's estates. An application was made by the committees of Sir Gregory's person to have an increased allowance made by the Court of Chancery, and 800*l.* a year more was granted, 500*l.* being for a carriage and horses. After Sir Gregory's death a caveat was entered against the will, which, however, Mr. Fryer withdrew. Witness had paid Rook his salary in the same manner after the death of Sir Gregory, first by the direction of Lady Turner, and, after her second marriage, by the order of Sir Edward Turner. The reason of the salary being continued was, that Rook had waited upon Sir Gregory for twenty years. Witness had taken Mrs. Neale to make an affidavit as to the state of Sir Gregory's mind at the time when there was no particular proceeding pending which required it to be made.

Re-examined by Mr. Rickards.—Mr. Fryer had the living of Eltham, in Kent, given him by the Lord Chancellor.

Mr. Henry Elston examined by Mr. Chambers.—This witness stated that he was clerk to Mr. Maberly, and corroborated the evidence given by Mr. Maberly as to the state of mind of Sir Gregory Page Turner on the different occasions when he called on Mr. Maberly respecting the making of his will. This witness also stated that he was attesting witness to the will of Sir Gregory, and that on the occasion of its being executed, he asked Sir Gregory if he should read it over to him, upon which Sir Gregory said, "No, thank you, Elston, you never knew me sign anything I had not read. The will is in accordance with my wishes." In the opinion of this witness when Sir Gregory executed the will he was perfectly in his senses.

Cross-examined by the Solicitor-General.—Between the years of 1826 and 1840, Sir Gregory always appeared to witness to be capable of transacting business. Sir Gregory was, at the time he came out of the Queen's Prison, which was about the year 1826, capable of understanding the contents of any document that might have been submitted to him.

Re examined by Mr. Chambers.—Before the year 1840, the state of Sir Gregory's mind was not so good as it was after. In 1841, Sir Gregory improved in bodily health, but was not so well in August, 1842, the last time when witness saw him.

By Lord Campbell.—Sir Gregory never executed any instrument that witness was aware of between the year 1823 and the time of his death, with the exception of the will.

Dr. Henry Herbert Southey, examined by Mr. Peacock.—Witness was a physician. Witness had been in practice about forty years. During that time witness had seen a great many insane persons. Witness saw Sir Gregory Page Turner about the year 1840. Witness saw Sir Gregory with the late Dr. Warburton. Witness was called in to see him to give an opinion about his case. In 1841 witness was called in to give an opinion respecting the removal of restraint. In 1840 witness considered Sir Gregory's rather a singular case; because, although insane in conduct, his conversation was not such as to infer insanity. His insane conduct was peculiarity in dress, dirty habits, and a disposition to hoard up things of no value. Sir Gregory discoursed rationally. In the year 1841 witness saw Sir Gregory twice. On those two occasions he conversed rationally. Dr. Warburton and witness thought it expedient to remove restraint, and to see how Sir Gregory behaved himself. At that time witness thought Sir Gregory could understand a will, and express his wishes in a rational manner as to the disposition of his property.

Cross-examined by Sir F. Kelly.—In 1841, when witness saw Sir Gregory twice, he could not say that he was of sound mind. In 1841 witness could not have recommended the commission to be superseded. There never was any time at which witness could have recommended the commission against Sir Gregory to be superseded. Witness would not have become attesting witness to his will, because Sir Gregory was under a commission of lunacy. The restraint was removed from Sir Gregory because the presence of the keeper annoyed him. The two conversations between Sir Gregory and witness, in 1841, lasted about half an hour each time. Sir Gregory used to drop tallow from the candle going up the staircase, and hoard up worthless old clothes. He also cut holes in his sheets. Those facts were proofs of insanity. Sir Gregory was also of generally dirty habits. He used to collect scraps of paper, and to fill his pockets with papers. Sir Gregory becoming affected with epileptic fits would lead witness to believe that insanity was not disappearing. The epileptic fits would lead witness to think that there was an injurious alteration in the structure of the brain.

Re-examined by Mr. Peacock.—In 1841 witness saw no vestiges of paralysis about Sir Gregory, nor at that time had he had any epileptic fits. In 1841 witness thought Sir Gregory capable of understanding the nature of a will. He was capable in 1841 of knowing the members of his family. In 1841 there was no defect in his memory that could be noticed.

By Lord Campbell.—If witness had been called in in 1841 to see Sir Gregory for the purpose of witnessing his will, he should have refused to do so, although no commission of lunacy existed, because of Sir Gregory's previous history.

Michael Rook examined by Mr. Rickards.—Witness was one of Sir G. P. Turner's keepers. Witness was appointed keeper in November, 1823. Witness attended Sir Gregory as a keeper until February, 1841. During that period witness constantly attended upon Sir Gregory Page Turner. Witness walked and drove out with him, and slept in his room. Witness used to converse with him, and knew all that he did. Witness left him when the personal restraint was removed in February, 1841. Witness returned to attend upon Sir Gregory in November, 1841. Witness then at first only slept in Sir Gregory's bed-room, as he had fits in the night, but left him in the day. That continued for about six months, but afterwards witness attended Sir Gregory by day as well as by night. The nature of witness's attendance was not the same at that time as it had been before February, 1841. Before February, 1841, witness considered Sir Gregory was under his care as an insane person, but after that period only on account of his fits. Witness remembered Sir Gregory having a carbuncle in his back in 1840. He got well from that in two months. About that time his mind appeared to improve, and he seemed more anxious about his family. About that time he left off some of his peculiarities. He used to rub his face with brown paper after he had washed it, but about the year 1840 he ceased to do so. Sir Gregory said he could leave all his peculiar habits off when he thought proper. About that time he also left off the habit he had of not opening a door with his naked hand. He was also willing to see his medical men. All the habits witness had mentioned had been entirely left off by Sir Gregory in February, 1841. None of those habits returned in November, 1841, when witness went back to Sir Gregory, and witness never knew them return. In February, 1841, witness considered Sir Gregory quite sensible and capable of transacting any business. He then conversed rationally. His behaviour and conduct were proper

Sir Gregory had an extraordinary memory. His conduct was prudent with respect to money matters. He always laid out his money to the best advantage, and used to buy books before witness left him in February, 1841. Sir Gregory was fond of old books. Witness observed towards the end of Sir Gregory's life that the fits had impaired his bodily health. Witness first observed that about the latter end of 1842.

William Holmes examined by Mr. Chambers.—Witness was the proprietor of the house in the Alpha-road where Sir Gregory Page Turner went to live. He came there in January, 1828. In April, 1829, witness was appointed by the Court of Chancery one of his keepers. Rook and witness attended him in April, 1840. In that month witness left Sir Gregory. In 1840 Sir Gregory's mind had improved. It was by Dr. Warburton's direction that witness left. In October or November, 1841, witness by accident met Sir Gregory in the Alpha-road. He was alone, and spoke to witness. Sir Gregory said, "Ah, Holmes, how do you do?" and shook hands with witness. Witness said to Sir Gregory, "I see you are a free agent." He said, "Yes, Holmes, and I hope I always shall be; and I hope you are comfortable in your situation. How is the duke?" (meaning the nobleman witness was then with.) Sir Gregory's manner had altered, and seemed more rational and less excited than it had been. Sir Gregory called upon witness in the autumn of 1842. He was in his carriage, out of which he got, and walked up and down the road with witness. Sir Gregory talked with witness on that occasion for about half an hour. His conversation was then rational. Rook was in the carriage. Witness said to Sir Gregory, "I see Rook is with you again." Sir Gregory replied, "I had a wish for Rook to come back again because I am not so well in my bodily health." On that occasion Sir Gregory shook hands with witness. Witness did not see anything in Sir Gregory's manner that rendered Rook's attendance necessary as regarded Sir Gregory's state of mind.

Cross-examined by Sir F. Kelly.—The conversation of Sir Gregory was always rational, with the exception of the first three months that he was at witness's house.

Mr. William Archer, examined by Mr. Peacock.—Witness was a surgeon, living in Montague-street, Portman-square. In 1841, witness attended Sir G. P. Turner. Witness first saw him in the November of that year. The late Dr. Warburton was then his physician. On the first occasion when witness saw Sir Gregory, he had burnt his hands in putting out Lady Turner's cap, which had caught fire. Lady Turner's hands were also burnt. Witness attended Sir Gregory a month or five weeks for the burns on his hands. Witness attended Sir Gregory about Christmas, 1841, on account of his having had an epileptic fit. Witness continued to attend from that time up to the time of his death. Witness visited him very frequently. The late Dr. Warburton continued to attend upon Sir Gregory up to the time of his death. Witness had frequent conversations with Sir Gregory. In 1841 Sir Gregory was quite rational, and had no delusions. After the fits came on, Sir Gregory's mind became more torpid. That was after he had had the fits for some time. Towards the end of 1842 there was more difficulty of making Sir Gregory understand, but when roused he understood what was said to him.

Cross-examined by the Solicitor-General.—Sir Gregory used to express great fondness for his daughter, and a wish to see her. Witness was present when Sir Gregory saw his daughter, and he received her with kindness. Witness never discovered that Sir Gregory had any delusions. Witness did not specifically try to discover the delusions, but conversed on general subjects.

By Lord Campbell.—In the end of 1841 or the beginning of 1842, if witness had been asked to attest the will of Sir G. P. Turner, he would certainly have done so.

The Rev. John Vaux Moore examined by Mr. Chambers.—Witness was the rector of Aspley Guys, in Bedfordshire, and was a first-cousin of the late Sir Gregory Page Turner. Witness saw him in the Alpha-road. In July, 1841, Sir Gregory paid witness a visit at his own house in Bedfordshire, and spent the greater part of the day with witness. He conducted himself and talked quite as other people would do. Sir Gregory was, in the opinion of witness, then in a sound state of mind and able to do a business act.

Cross-examined by Sir F. Kelly.—Witness never transacted any business with Sir G. P. Turner. Witness never resided with Sir Gregory. The conversation was upon general topics.

Miss Frances Ann Moore, examined by Mr. Peacock.—Witness was a cousin of the late Sir G. P. Turner, and was sister to the last witness, and lived with him. Witness had known Sir G. P. Turner from a boy, and he was always a person of eccentric habits. He was rational in talking. Witness could not recollect whether she had ever seen Sir Gregory do anything irrational.

Cross-examined by the Solicitor-General.—Whenever witness saw Sir Gregory, he was rational in his conversation at all times. Sir Gregory never in any manner, accidentally or otherwise, annoyed witness.

Re-examined by Mr. Chambers.—When witness saw Sir Gregory in the Alpha-road, he conversed quite rationally.

Miss Mary Durrosi examined by Mr. Rickards.—In 1832 witness went to reside as governess in the family of Lady Winston Barron, who was Sir G. P. Turner's sister. Witness remained five years in the family of Lady Barron. After witness left, the intercourse was kept up with the family of Lady Barron. Witness visited Sir Gregory Page Turner with Lady Barron, and also after she left. Those visits continued up to his death. When witness saw Sir Gregory his conversation was rational. Witness used to take Lady Barron's children to see him, and he was glad to see them. Witness visited Sir Gregory in the Alpha-road, Montagu-square, and Gloucester-place. Witness always found his conversation rational.

By the Jury.—Witness played with Sir Gregory on Twelfth Night in Gloucester-place, at three-handed whist. Sir Gregory played very well, and gained twice, and witness lost 2*s*. (Laughter.)

Mr. Robert Hare examined by Mr. Chambers.—Witness had been fifty years a clerk at Messrs. Coutt's bank. Witness saw Sir G. P. Turner. Witness saw him one day at the banking-house in the autumn of 1841. He came to return a visit witness had paid him in the Alpha-road. He stayed only a few minutes, and conversed rationally. In the latter part of June, 1841, witness had seen Sir Gregory in the Alpha-road. Witness's wife had been invited to dine, and witness joined Sir Gregory and Lady Turner at dessert. Witness was with Sir Gregory on that occasion for three or four hours. He then conversed rationally, and behaved as a gentleman.

Mrs. Hare examined by Mr. Chambers.—Went to see Sir Gregory in June, 1841, being an old friend of Lady Turner's. Witness saw Sir Gregory four times. On the first occasion, when witness saw him, he conversed very rationally, and in a gentlemanly manner. On the other three occasions Sir Gregory conducted himself as a rational man would do.

Miss Dryden examined by Mr. Peacock.—Witness was the daughter of the late Sir Henry Dryden. Witness went with Mrs. Fryer to see Sir G. P. Turner. Witness went twice. On both occasions witness stayed for about four hours, and Sir Gregory discoursed rationally.

The Rev. Lempster Dryden examined by Mr. Rickards.—Witness was the cousin of late Sir G. P. Turner, and visited him in the Alpha-road, twice in March, 1841. Witness, on the second occasion, spent the whole evening with Sir Gregory, who was in a good state of mind. He conversed most rationally. His manner was calm, like that of an ordinary person. Witness thought that at that time Sir Gregory was competent to transact any business of life.

Cross-examined by the Solicitor-General.—Witness only saw Sir Gregory two or three times in twenty years, and had been one of the committees of his person. Witness, however, left the whole business to Lady Turner and Mr. Maberly.

We should state that this witness was the plaintiff in the cause, but he was authorized to be made a witness by the direction of the Court of Chancery.

With this witness's evidence the case for the plaintiff closed.

The Solicitor-General then proceeded to address the jury. He said the present case was certainly one of the most extraordinary that had ever come into a court of justice, for in this case, here was the extraordinary fact of a gentleman who had a commission of lunacy hanging over his head, going to the solicitor to the commission, and executing a will prepared by him, and unknown to any one but Mr. Maberly and his clerk. (The learned Solicitor-General here read the judgment of the late Vice-Chancellor of England, in which he said that the story with regard to the will was one which required investigation.) In that opinion he (the Solicitor-General) agreed. It was admitted by Mr. Maberly and Lady Turner that Sir Gregory Page Turner had been insane up to 1840, whilst some of the plaintiff's witnesses said that he was a rational man at all times. There was, however, a fact which proved that Lady Turner and Mr. Maberly thought Sir Gregory was insane before 1840, for when in the year 1838 his daughter was about to be married, no intimation of that fact was given to the poor lunatic. Surely, if he had not been insane, would not so interesting a fact to a father as his daughter's marriage have been communicated to him? His learned friend (Mr. Chambers) admitted that Sir G. P. Turner had been once insane, but said, as bodily health declined his mental state improved. He (the Solicitor-General) expected that some curious medical evidence would have been offered to prove such a singular theory, but it had not. With respect to Lady Turner's evidence, it must be received with great caution; because if the will were established, she got Battlesden House for life, 300*l.* in money, and other property. Mr. Maberly had also an interest in establishing the will, for if it should not be established, the management of some of the estates would pass into the hands of Mr. Fryer, and Mr. Maberly would cease to be employed as the solicitor to the estates. If Sir G. P. Turner had not been insane in 1841 and 1842, would he who had estates in Bedfordshire, Oxfordshire, and Kent, have been treated in such a manner as only to be allowed a little pocket-money? With regard to the will, he (the Solicitor-General) asked the jury to look at the will, and to see whether these alterations said to have been made by Sir G. P. Turner, had not been actually made by Mr. Maberly himself. It was not likely that Sir Gregory would have made a will such as he had done, depriving Mr. Charles G. Fryer of his just rights, if he had been in a sound state of mind. In the course of the cause the name of William Paxton had been mentioned as having had the greatest influence over the mind of Sir G. P. Turner, and who would therefore have been a most favourable witness for the plaintiff; yet he had not dared to do so. It was not to be believed that Sir Gregory Page Turner would have put a clause in his will by which, if his daughter disputed the will, he would reduce the 2000*l.* a year to 300*l.* a year. This argument derived strength from the fact that every witness for the plaintiff had represented Sir G. P. Turner as being very fond of his daughter. With regard to Sir Gregory being rational in his conversation, that was no proof that he was not insane, because there were many people who had the germs of insanity in their minds who were able to converse in a perfectly rational manner. He (the Solicitor-General) would prove that if ever a man was mad, Sir G. P. Turner was at the time he made the will. He would prove that Sir Gregory could not be got to change his linen when Rook left him. It would be proved that the first night after the restraint was withdrawn from him he came home at ten o'clock quite drunk. He (the Solicitor-General) would prove that Sir Gregory went for a month without having a clean shirt, and then the clean one was put on over the dirty one. He would show that he cut holes in his sheets and went to bed in his clothes. He (the Solicitor-General) would prove that Lady Turner used to hold out as a threat, to induce Sir Gregory to do anything, that if he did not do it, the commission could not be superseded. He would prove that Lady Turner and Mrs. Neale schooled Sir G. P. Turner about his peculiarities—such as his fear of infection by shaking hands with his naked hand, his dislike to dogs and cats. They used to tell him if he did not leave them off, the commission could not be superseded. The landlady of the hotel at Luton, to which Sir Gregory went, would prove that he was quite childish. There would be evidence given as to strange proceedings which took place whilst Sir Gregory was at Brighton. One night he got up in the middle of the night, and, with John, his page, both parties being in their night-shirts, went into the bedroom of Mrs. Graves, who had gone to Brighton with him in place of Lady Turner. Sir Gregory then compelled Mrs. Graves to sit up in bed, and to read a letter which had been sent him from Lady Turner, three times over. The learned Solicitor-General then concluded a speech of two hours' length by calling on the jury to uphold the commission of lunacy which had been issued in 1823, unless they saw good ground for setting it aside.

Eliza Long examined by Mr. Barstow.—Witness was formerly in the service of Mr. Holmes at the house in the Alpha-road, where Sir G. P. Turner lived. Witness was afterwards in Rook's service when he took the house, and continued in the service after Lady Turner came to live in the house. Witness stayed in the service until two months after Rook left, which he did in February, 1841. Witness had been in the habit of seeing Sir Gregory for three years and nine months. Witness had seen Sir Gregory in the streets with his keepers when a funeral passed, and Sir Gregory would cross to the other side of the way. When witness lived in service in the Alpha-road, she considered Sir Gregory different to other gentlemen. He was frightened at dogs and cats. Once Sir Gregory had a new suit of clothes, and Mr. Holmes's dog licked one of Sir Gregory's gaiters, and he never would wear it again. Rook used to shave Sir Gregory every other day. He did not like to be shaved, and asked to be excused. Sir Gregory used to eat very greedily. His clothes were very dirty and greasy. He used to put his clothes inside his bed frequently. One leg of Sir Gregory's trousers was always turned up when he came down from his bedroom in the morning. It was always turned up after he went up to dress. Sir Gregory was dirty in his person. Sir Gregory never washed his face clean, and Rook sometimes washed it for him. Sir Gregory would never touch a towel if it had touched the floor. He would not allow his bedclothes to touch the floor. Witness made his bed, and Sir Gregory told her not to let the bedclothes touch the floor. He put his sheets underneath the bed, and slept in the blankets. He used to tear the bed furniture, and then sew it up. Every other Sunday was Sir Gregory's needlework day. Sir Gregory would not shake hands with people. He used to fill his trouser's pockets with papers. He was very careful of the papers, and additional buttons were put on his pockets to secure them. Sir Gregory had a newspaper every morning, but he did not read it till the next day, in order, as he said, that it should get properly dried. The newspaper was the *Morning Herald*. On the first day, when Sir Gregory particularly desired to know the news, some one held the newspaper while he read it. Sir Gregory would let his dinner stand for a quarter of an hour or twenty minutes before he would eat it. He had dinner alone. If a knife or fork fell to the ground Sir Gregory would not use it. He would not let it be picked up till another was brought. He had a great objection to medical men. He used to call Dr. Southey "the lawyer." Witness had seen Mr. Maberly at Alpha-road. Sir Gregory called Mr. Maberly "the tailor."

Cross examined by Mr. Chambers.—Witness had fits herself when Rook was at the Alpha-road. The fits were epileptic fits. There was a mistletoe in the kitchen in the house at Alpha-road, and Rook's son tried to kiss witness, and she threw herself back and became ill.

Mrs. Frances Freeman examined by Mr. Rowe.—Sir Gregory had peculiar habits; he used to open the doors with the skirts of his coat, which he took in his hand. (Here the witness corroborated many of the facts deposed to by the witness Eliza Long.)

Anne Watson examined by Mr. Barstow.—Witness was now lady's-maid to Mrs. Fryer, the daughter of Sir G. P. Turner. Witness was lady's maid to Mrs. Fryer when at Cheltenham. Witness went for three months to reside with Lady Turner in the Alpha-road. Sir Gregory never acted as the master of the house. He put his clothes in his bed. Witness slept in the room in which Eliza Long had slept, which was next to Sir Gregory's room. (This witness also stated similar facts regarding Sir Gregory's habits as former witnesses had done. She also gave similar testimony to that given by Eliza Long respecting the habit which Sir Gregory had of disturbing the person at night who slept in the next room to him.)

Mrs. Elizabeth Buclin examined by Mr. Rowe.—Witness was a widow keeping an inn at Luton. In 1841 Sir G. P. Turner came with a large party to witness's inn. Mr. William Paxton was one of the party. Sir Gregory and party stayed about three weeks. He had a fit one Sunday, which was very violent.

Mary Maxfield examined by Mr. Barstow.—Witness had been lady's-maid to the late Mrs. Graves, who was first cousin to Sir G. P. Turner. Mrs. Graves visited Sir Gregory when he lived in the Alpha-road. Witness used to see Sir Gregory, who was not like other people. In October, 1841, Mrs. Graves and Sir Gregory went down to Brighton. Witness also went down. Mr. William Paxton was likewise one of the party. Mrs. Graves and Sir Gregory dined together. The doctors allowed Sir Gregory seven glasses of wine, and he would have that quantity of wine measured out several times over. One Saturday a letter came from Lady Turner, which was read. In the night Sir Gregory and John, the page, with a light, came into Mrs. Graves's room in their night-clothes. Witness slept in Mrs. Graves's room. When Sir Gregory came in he asked which was Mrs. Graves, who answered, "I am Mrs. Graves." Sir Gregory had Lady Turner's letter in his hand, and said he wished to have "Lady Page's" letter read to him. By Lady Page he meant Lady Turner. Witness got out of bed and held a light at the foot of the bed, so that Mrs. Graves might read the letter. John, the page, remained at the door, and Sir Gregory stood at the side of the bed. Mrs. Graves read the letter three times over, and Sir Gregory, when it was first read, asked witness if it had been correctly read. Sir Gregory and the page then went back to bed. Sir Gregory used to walk about in the night. John, the page, walked about with him, and Sir Gregory talked to him. On another occasion Sir Gregory came in the middle of the night and knocked at Mrs. Graves's bedroom door. Mrs. Graves got out of bed to him, and he said John had stolen all his things. Mrs. Graves told him to go to bed, and he called John "a d—d vagabond." One evening, Sir Gregory removed the sofa from the sitting-room into his bedroom, and laid down upon it. Witness sat by his side in a chair. Sir Gregory opened the window, and said when the window was opened the door must be shut. He then shut the window and opened the door. He did so three times. He then asked witness to sing. (Laughter.) Witness sang to Sir Gregory. (Renewed laughter.) He then said, "That will do—that will do." Witness then went away, because she was afraid.

Stephen Mackay examined by Mr. Rowe.—Witness had been engaged since 1839 in attending upon lunatics. Witness was attached to the late Dr. Warburton's establishment at Hoxton. Witness was sent to attend on Sir G. P. Turner, when he lived in Gloucester-place. Witness first attended upon Sir Gregory in June, 1842, to relieve Rook for one day. Sir Gregory was pleased with witness at first, but afterwards wished to have Rook back again the same day. At that time Sir Gregory was almost deprived of his speech by paralysis. He expressed himself by hallooing. Sir Gregory did not exercise any free-will about any matter, and was incapable of taking care of himself. At that time he had no command of money. Witness was present at his death. He died in an epileptic fit.

Cross-examined by Mr. Chambers.—It was not a very violent fit in which he died, but nature was quite worn out.

Mrs. Beecher examined by Mr. Barstow.—Witness was the wife of Captain Beecher, R.N., who knew Captain Bayfield, the brother of Lady Turner. In 1840 witness and her husband took tea with Sir Gregory and Lady Turner in the Alpha-road. Witness considered Sir Gregory at that time to be an imbecile person. He merely bowed in answer to observations. He could not keep up a conversation. His speech was thick.

Dr. James Sutherland examined by Sir F. Kelly.—Witness was physician to St. Luke's Hospital for thirty years, and began to practise in 1805. Witness had now retired from practice. Witness had given his attention particularly to lunatic cases. Witness first saw Sir G. P. Turner on the 6th of December, 1823. Witness was called in to oppose the commission of lunacy. Witness saw Sir Gregory in the Queen's Prison. Sir Gregory had three physicians called in to oppose the commission, and witness was one of them. Witness examined Sir Gregory three times; the result of that examination was, that he (the witness) believed that Sir Gregory laboured under delusions. The chief delusion was his fear of infection. He used to cut holes in linen. Sir Gregory said that his son, who was dead, had been murdered by a Mr. Green, from his not putting restoratives into his mouth when he was dead, which he (Sir Gregory) had desired him to do. Sir Gregory was reminded that the child's head had been examined by Sir Astley Cooper, upon which he became angry, and said his child had been murdered. In 1824, by the order of the Lord Chancellor, witness saw Sir Gregory three times in July. His disease had not improved at all. Witness saw him three times in 1825, and he was still the same. Witness saw him also in 1826, and he was then no better. One of his delusions was that his family had conspired against him, and he said his brother, Sir Edward Page Turner, was illegitimate. Where a person was afflicted with an insanity like that of Sir Gregory's, and where it had continued for so long a time as it had in his case, the chances were against his recovery. Witness knew of no instance of a person who had been insane for so long a time as from 1823 to 1839, recovering so as to be fit to make a will. Where epilepsy and paralysis came on they were likely to prevent insanity disappearing. In order to try whether a person had recovered his reason, it was necessary rigidly to test the patient on the subject of the delusions he had laboured under when insane. The fact of Sir G. P. Turner talking rationally on some subjects, would not be a proof that he was sane, unless his mind was free from delusion. Witness thought Sir Gregory was not, when he knew him, in a fit state of mind to make a will. Witness never knew of an instance of insanity, followed by paralysis and epilepsy, disappearing, when the insanity had existed for many years. The rule at the hospitals was to give mad patients twelve months to recover, and after that time, if they did not improve, they were discharged as incurable. Witness had heard the evidence of Dr. Southey, but it had not altered his opinion with regard to the case of Sir G. P. Turner.

By the jury.—In a case like Sir Gregory's there were not likely to be lucid intervals.

Dr. Conolly examined by Mr. Barstow.—Witness had been a physician twenty-eight years, and had been physician to the Hanwell Lunatic Asylum eleven years. If a person had been insane for fifteen years, epilepsy supervening would make the disease worse. In such a case witness had never known a person cured.

This was the case on behalf of the defendant.

Mr. Chambers then replied in a speech of considerable length, and of great eloquence and ability.

The Lord Chief Justice (at six o'clock) proceeded to sum up the case. He said that this case was an issue directed by the late Vice-Chancellor of England, to try the validity of a will, executed by the late Sir Gregory Osborne Page Turner, on the 15th of June, 1841. The validity of that will depended upon the opinion the jury should form of the state of Sir Gregory's mind at the time it was executed. If it had been clearly and satisfactorily proved to them that at the time the will was made Sir Gregory Page Turner was of competent understanding, that he knew its contents, and was perfectly aware of what he was doing, and if he had a disposing mind, then the will would be a valid one, notwithstanding the intellect of Sir Gregory was subsequently impaired, and the fact that a commission of lunacy was in existence. Whether he was in a proper state of mind on the 15th of June, 1841, was entirely for the jury to say. The Lord Chief Justice then went entirely through the evidence, making observations upon it as he proceeded.

His lordship concluded his summing up at twenty minutes past nine o'clock, and the jury immediately returned a verdict for the plaintiff.

